# Statistical Inference for Hardy-Weinberg Proportions in the Presence of Missing Genotype Information

**DOI:** 10.1371/journal.pone.0083316

**Published:** 2013-12-31

**Authors:** Jan Graffelman, Milagros Sánchez, Samantha Cook, Victor Moreno

**Affiliations:** 1 Department of Statistics and Operations Research, Universitat Politècnica de Catalunya, Barcelona, Spain; 2 Biomedical Research Unit, Vall d'Hebron Research Institute, Barcelona, Spain; 3 Financial Network Analytics, Barcelona, Spain; 4 Cancer Prevention and Control Program, Catalan Institute of Oncology (ICO), Biomedical Research Institute of Bellvitge (IDIBELL), Consorcio de Investigación Biomédica de Epidemiología y Salud Pública (CIBERESP), and School of Medicine, University of Barcelona (UB), Barcelona, Spain; Universitat Pompeu Fabra, Spain

## Abstract

In genetic association studies, tests for Hardy-Weinberg proportions are often employed as a quality control checking procedure. Missing genotypes are typically discarded prior to testing. In this paper we show that inference for Hardy-Weinberg proportions can be biased when missing values are discarded. We propose to use multiple imputation of missing values in order to improve inference for Hardy-Weinberg proportions. For imputation we employ a multinomial logit model that uses information from allele intensities and/or neighbouring markers. Analysis of an empirical data set of single nucleotide polymorphisms possibly related to colon cancer reveals that missing genotypes are not missing completely at random. Deviation from Hardy-Weinberg proportions is mostly due to a lack of heterozygotes. Inbreeding coefficients estimated by multiple imputation of the missings are typically lowered with respect to inbreeding coefficients estimated by discarding the missings. Accounting for missings by multiple imputation qualitatively changed the results of 10 to 17% of the statistical tests performed. Estimates of inbreeding coefficients obtained by multiple imputation showed high correlation with estimates obtained by single imputation using an external reference panel. Our conclusion is that imputation of missing data leads to improved statistical inference for Hardy-Weinberg proportions.

## Introduction

The Hardy-Weinberg principle [1], [2] states that the genotypes AA, AB and BB at a diallelic locus with alleles A and B will occur with relative frequencies 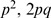 and 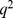 respectively, where 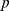 is the allele frequency of A and 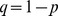. In the absence of disturbing forces (drift, mutation, selection, migration, etc.) the Hardy-Weinberg proportions (HWP) are achieved in one generation of random mating. If disturbing forces remain absent then allele and genotype frequencies will no longer change, a condition referred to as Hardy-Weinberg equilibrium (HWE). Several statistical procedures are available to test if observed genotype counts are compatible with the theoretical HWP. These tests are often called “tests for HWE”, though strictly speaking they do not test equilibrium (stable allele and genotype frequencies) but test if sample genotype counts are in agreement with HWP. For this reason, we refer to these tests as tests for HWP in the remainder. Till recently, the classical 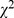 test was the most popular way to test for HWP [3], though nowadays the exact test is more popular [4] and other alternatives have been proposed [5]. Statistical tests for HWP play an important role in genetic association studies. HWP tests are helpful for the detection of genotyping errors [6]–[8] and can also be indicative of marker-disease associations when disequilibrium is detected among affected individuals [9]–[12]. For these reasons, databases of genetic markers are usually tested for HWP before or after their use in association studies.

**Figure 1:**
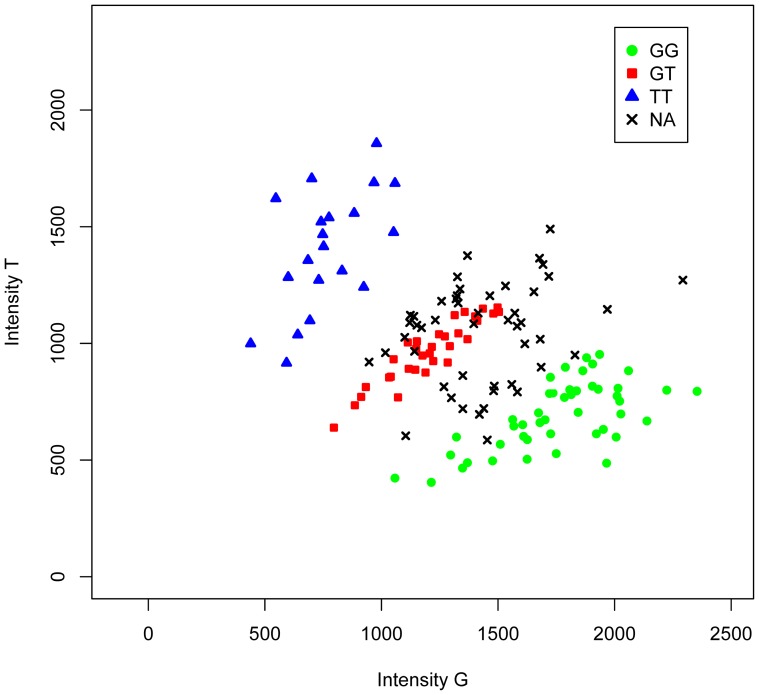
Intensity plot of a G/T polymorphism for 146 individuals. Missing values (NA, 33% of the data) indicated by black crosses occur mainly at the boundaries of homozygotes and heterozygotes.

The occurrence of missing data is a common problem in genotyping studies. Genotype calling algorithms assign the genotype (AA, AB or BB in a generic notation) of an individual on the basis of the A and B allele intensities by means of a clustering/classification algorithm. The latter algorithm assigns a missing outcome to an individual if it is unable to find an appropriate genotype given the observed allele intensities. Often missing outcomes (NA) occur at the frontiers of the clouds of homozygotes and heterozygotes in a plot of allele intensities as shown in Figure 1.

It is not uncommon to have 10% or more missing genotype information in a genomic database. However, the percentage of missing values may strongly vary from one marker to another. For a particular marker, 0 through 100% of the information may be missing. When markers are tested for HWP, the missing genotype information is often discarded. Discarding missing values brings about two problems. First of all, due to a reduced sample size, power for detecting disequilibrium will decrease. Second, if the genotype information is not missing completely at random, then the statistical inference regarding HWP may be biased.

In this paper we focus on the potential bias in statistical inference about HWP. We do this by comparing the inference made by discarding missing values with the inference made by imputing missing values, thereby using Rubin's multiple imputation approach [13]. For the imputation of missing values we propose to use statistical models that use information from both allele intensities and/or neighbouring markers. The structure of the remainder of this paper is as follows. In the section Methods we outline how principles of missing data analysis apply in the context of diallelic genetic markers. In the Results section we show examples of statistical inference for HWP of single nucleotide polymorphisms (SNPs) with missing data, and compare multiple imputation with single imputation based on a reference panel. We finish with a Discussion section and supply software that can perform statistical tests for HWP in the presence of missing genotype data.

## Materials and Methods

In this section we discuss basic principles of missing data analysis in the context of diallelic genetic markers, and consider the missing data mechanism and missing data imputation.

### Missing data mechanism

The statistical theory on missing data distinguishes three types of missing data mechanisms [13]. We briefly outline these embedded in the genetic context. Genotype data for a particular SNP may be missing completely at random (MCAR). In this case, the observed genotypes constitute a random sample of a (hypothetical) data set of completely observed individuals. If the data is MCAR, then testing for HWP by simply discarding the missing observations is not too problematic. It only entails a loss of power for detecting deviations from HWP because the sample size is smaller. Alternatively, genotype data for a SNP may be missing at random (MAR). Under a MAR mechanism, the probability that a genotyping result is missing for a particular SNP may depend on the observed data (e.g. allele intensities or other SNPs) but, conditional on the observed data, may not depend on the values of the SNP itself. Finally, the data may be missing not at random (MNAR), meaning that the probability of a missing genotype result does depend on the values of the SNP under consideration, even after controlling for the relationships of this SNP with allele intensities and other SNPs. Whether genotype data can be considered MCAR can be investigated to some extent. Under MCAR, the allele intensities are expected to be, on average, the same for individuals with a missing genotype as for individuals with observed genotypes. This can be assessed by comparing average allele intensities with a Student 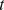 test. Two 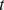 tests can be performed for each marker, one for each allele intensity (A and B). The two allele intensities (A and B) can also be compared jointly for missings and non-missings by testing equality of mean intensity vectors with Hotelling's 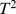 statistic. Examples are given in the Results section. Statistical testing can discard the MCAR hypothesis, though this does not necessarily imply that the MAR assumption will hold. The MAR hypothesis is often assumed, and considered reasonable if important predictors of the SNP with missings are included in the imputation model [13]. Besides allele intensities, genotyping results of (correlated) neighbouring markers are often available. Under the MCAR assumption, the distribution of the genotypes at such neighbouring markers is supposed to be the same for missing and non-missing observations for the SNP under consideration. In this context, the MCAR assumption can be tested by chi-square or exact tests on contingency tables of genotype counts.

### Missing data imputation

A statistical test for HWP can be viewed as a hypothesis test for a disequilibrium parameter. In this paper we use the classical inbreeding coefficient (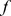) as a measure for disequilibrium. The term inbreeding coefficient may be regarded as a misnomer, since in our work we imagine observed disequilibrium to arise from genotyping error or by chance, and not from inbreeding. However, we maintain the term “inbreeding coefficient” for historical reasons and because of its widespread use in population genetics. The degree of disequilibrium can be parametrized by using the inbreeding coefficient 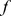 [14], and under this parameterization, the population genotype frequencies are given by 
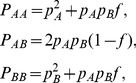
(1)with 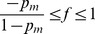, where 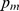 is the minor allele frequency 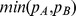. If 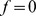 then the genotype frequencies correspond to the Hardy-Weinberg proportions. For 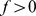 there is heterozygote dearth, and for 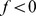 there is heterozygote excess. Parameter 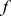 can be estimated by maximum likelihood (ML) using the multinomial distribution. The ML estimator and its variance [15] are given by 
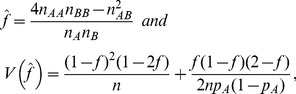
(2)where 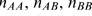 and 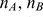 are the respective absolute genotype and allele counts, and 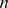 is the total sample size. To compute the variance of 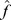, 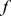 and 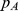 are substituted by their sample estimates. We note that the ML estimator is related to the classical chi-square statistic for HWP by 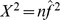. The genotyping results obtained for a particular SNP depend in the first place on the allele intensities, as the latter form the basis of the classification (see Figure 1). In order to impute missing data, we used multinomial logit models with different sets of predictors. We fitted the multinomial logit model [1]

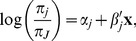
(3)where 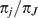 represents the ratio of the genotype frequency of the 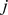 th genotype (with 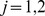) with respect to a reference genotype frequency 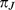. Usually the most frequent genotype is chosen as the reference genotype. E.g. if 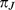 refers to the probability of a BB genotype, then the log-ratios of AA and AB with respect to BB are modeled as a function of a set of predictors 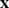 (here allele intensities and/or genetic covariates). The coefficients 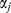 constitute the intercept terms of the model, and the coefficients 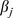 represent the change in log odds of being of a particular genotype for a one-unit increase in one predictor, other predictors held constant. The multinomial logit model, also known as polytomous logistic regression, is a particular case of a generalized linear model [16], [17]. The multinomial logit model generalizes logistic regression for a response variable with more than two outcomes. For many SNPs three genotypes are observed and therefore the multinomial logit model is the indicated model. For some SNPs only two genotypes are observed. With only two outcomes, the model is equivalent to logistic regression. The multinomial logit model was used in combination with multiple imputation by chained equations [18], the MICE algorithm. MICE allows one to specify an imputation model for each variable in the data set. The algorithm obtains the posterior distribution of the parameter of interest (inbreeding coefficient 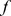 in this study) by iteratively sampling conditional distributions with a Gibbs sampler. MICE is apt for data sets that have a non-monotone pattern of missings, as is the case for SNP data, where missings of covariates are imputed as well. For more details on the MICE algorithm we refer to Van Buuren [19] and Van Buuren and Groothuis-Oudshoorn [18]. Multiple imputation yields a set of 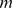 complete data matrices of genotype information. To finally be able to perform statistical inference for HWP, inbreeding coefficients and their variances are estimated for all imputed data sets, and these estimates are combined according to Rubin's pooling rules [13], [20]. In short, for 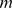 imputations the parameter estimates 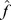 and their variances are combined by computing their means 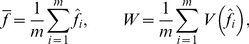
(4)where 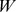 is called the average within-imputation variance. Next, the between-imputation variance (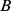) and the total variance (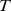) are then computed as 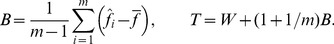
(5)


A test statistic for HWP (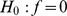) is then given by 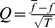. Under the null, this statistic has a 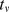 distribution with 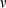 degrees of freedom, 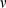 given by 
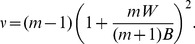
(6)


After imputation, a 95% confidence interval for 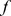 is given by 
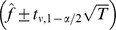
 and a *p*-value for a two-sided test for HWP is given by 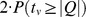. The sample inbreeding coefficient 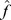 is an intraclass correlation coefficient. The normality of this coefficient can be improved by using Fisher's 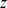-transformation 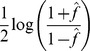, and this is recommended in general when combining correlation coefficients from multiple imputations into a single estimate [21].

### Dataset used as a practical application

To test the proposed methods in a real dataset, we have analyzed the data from 146 individuals (99 cases with colon cancer and 47 controls) that participated in a clinical study that aimed to identify cancer biomarkers. Written informed consent was obtained from all participants and the study protocol and consent forms were approved by the Bellvitge Hospital Ethics Committee. These subjects were genotyped with the Affymetrix Human SNP Array 6.0. For the analysis performed in this study, a 6 Mb genome region was selected. Data was anonymized and dereferenced while maintaining the correlation structure. All analyses were performed on secured servers under the supervision of the investigators to avoid accidental disclosure of the genetic data.

## Results

In this section we first describe the data set we will use for our study on HWP and missing genotype data. Secondly, we investigate whether the MCAR assumption is tenable for the genotype data. Next, we will give a detailed example of the use of multiple imputation for inference for HWP of a single SNP. Thereafter, we evaluate the consequences of using multiple imputation for the inference regarding HWP for the whole database. Finally, we compare our multiple imputation approach with results obtained by single-shot imputation using a reference panel.

### Description of the data

**Figure 2:**
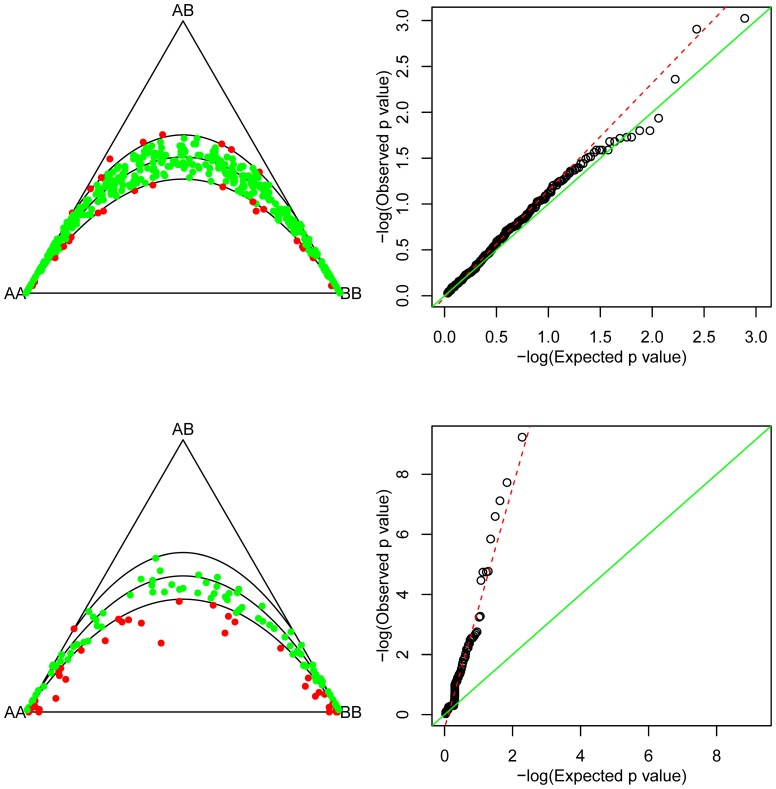
Ternary plots and Q-Q plots for Hardy-Weinberg proportions. Curves in the ternary plots indicate the HW parabola, and the limits of the 95% acceptance region of a 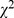 test for HWP. Top row plots are for 545 fully observed SNPs. Bottom row plots are for 140 SNPs with 10 to 50% missings (missings were discarded in these plots). The Q-Q plots show two lines, a solid 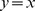 reference line and an estimate of the linear tendency in the cloud of points (dashed).

The database included 1685 SNPs selected from a 6 Mb genomic region with a median spacing of 1932 bp. Overall the data contained 3.5% missing data, though the degree of missingness per SNP varied from 0 to 100%. On a by-individual basis, the percentage of missings did not exceed 12% per individual, indicating good quality of the biological samples. 545 SNPs were completely observed. Allele intensities for A and B were always completely observed. We first tested completely observed SNPs and SNPs with 10 to 50% missings separately for HWP, using a chi-square test without continuity correction, and simply discarding missing genotypes. We did this graphically [22] by representing the SNPs in ternary plots and Q-Q plots, as is shown in Figure 2.

Figure 2 shows that the completely observed SNPs are in general in accordance with HWP, with 6% of the SNPs significant at the 5% level. The bottom row of Figure 2 shows SNPs with 10–50% missing data. Too many SNPs show statistically significant deviations from HWP (27%). The ternary plot shows that deviation from HWP is mainly due to a lack of heterozygotes. The *p*-value distribution of HWP tests is known to be non-uniform under the null hypothesis [23]. We note that the Q-Q plots shown in Figure 2 are made with respect to the truly null distribution of the *p*-values for the data set under study.

### The MCAR assumption

We first assessed whether the MCAR assumption is reasonable for the data. Testing the null hypothesis of equal mean allele intensities for missing and non-missing genotypes is possible only if a SNP has a sufficient number of missing observations. We therefore restricted this analysis to SNPs with 10–50% missing values, and this guaranteed a sample size of at least 15 observations for the missing observations. We tested the null hypothesis of equal mean intensities 
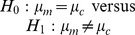
 for missing and non-missings genotypes for both intensities of each SNP separately, using univariate Student 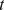 tests. We also used multivariate Hotelling 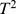 tests, both with and without assuming homocedasticity to compare the two mean vectors of intensities jointly (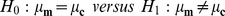). The number and percentage of significant results are reported in Table 1.

**Table 1:** Significance tests of equal mean intensities for missing and non-missing genotyping results.

Test	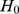	assumption	# significant	% significant
Student's 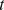 Allele A	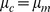	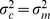	98	70.0
Student's 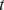 Allele B	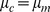	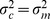	100	71.4
Student's 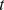 Allele A	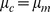	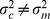	101	72.1
Student's 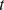 Allele B	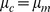	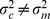	103	73.6
Hotelling's 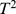	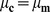	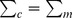	123	87.9
Hotelling's 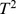	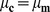	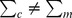	128	91.4

Number and percentage of significance tests are given for 140 non-monomorphic SNPs with between 10 and 50% missing values (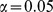). Results are given for tests with and without homocedasticity assumption (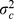 is the intensity variance of the completely observed genotypes, 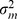 is the intensity variance of the missing genotypes).

Table 1 shows that the MCAR hypothesis is clearly not tenable for the data. If MCAR would hold, we expect to obtain, by chance alone, about 5% significant results, whereas we find 70–90% significant tests. Allele intensities are apparently different for observed and non-observed genotyping results.

We imputed missing genotype data using the statistical model described in the Methods section. We used the MICE package [18] to create imputed data sets. We first discuss the results for the one G/T polymorphism displayed in Figure 1 in the Introduction, and next consider the results obtained for the whole set of 140 SNPs with 10–50% missings.

### Multiple imputation of a single polymorphism

**Figure 3:**
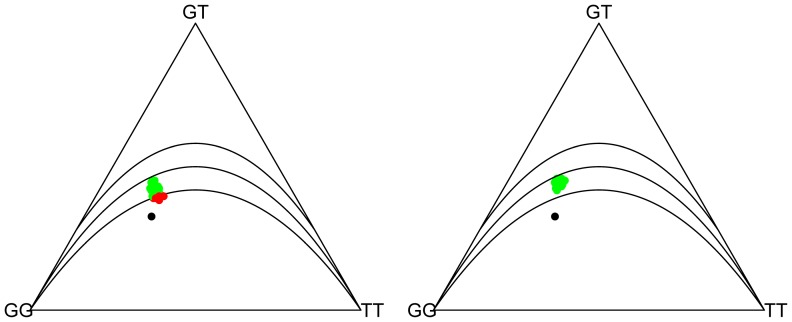
Ternary plots of *m* = 50 imputed data set for the G/T polymorphism of Figure 1. Curves in the ternary plots indicate the HW parabola, and the limits of the 95% acceptance region of a 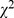 test for HWP. Left panel: imputed data sets with allele intensities as covariates (model 3). Right panel: imputed data sets with allele intensities and 1 covariate SNP (model 5).

The counts for the G/T polymorphism displayed in Figure 1 are given by (46,32,20,48) for GG, GT, TT and missings respectively. When missings are ignored a chi-square test (without continuity correction) for HWP gives 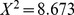 (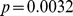), leading us to reject the null hypothesis of HWP. The estimate of the inbreeding coefficient is 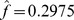. A two-sided exact test for HWP leads to the same conclusion (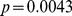). We performed multiple imputation using the models and pooling rules described in the Methods section. The effect of multiple imputation on HWP is illustrated for this SNP with 50 imputed data sets and two models in Figure 3. Imputation with the multinomial logit model and intensities as covariates leads to imputed data sets with slightly higher T allele frequencies and an increased number of heterozygotes (left panel). Most imputed data sets fall within the acceptance region of a test for HWP. Inclusion of a correlated covariate SNP further increases the imputation of heterozygotes, leading to imputed data sets that do no longer deviate from HWP (right panel). We considered several multinomial logit models for the imputation of the missings. Results for the estimation of the inbreeding coefficient with these models are shown in Table 2.

**Table 2:** Inbreeding coefficients, confidence intervals, *p*-values and missing data statistics (relative increase in variance (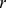), and fraction of missing information (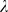)) for multiple imputation with different multinomial logit models, and for single imputation with IMPUTE2.

Model	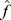	95% CI	*p*-value	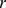	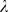
1. discarding NAs	0.298	(0.138,0.457)	0.003	-	-
2. random imputation	0.301	(0.117,0.485)	0.001	0.283	0.222
3. intensities	0.110	(−0.069,0.288)	0.229	0.200	0.167
4. 1 correlated SNP	0.059	(−0.107,0.225)	0.487	0.039	0.038
5. intensities + 1 SNP	0.060	(−0.109,0.228)	0.488	0.065	0.062
6. 10 correlated SNPs	0.060	(−0.106,0.225)	0.479	0.028	0.027
7. intensities + 10 SNPs	0.055	(−0.111,0.222)	0.516	0.044	0.042
8. IMPUTE2	0.023	(−0.140,0.185)	0.786	-	-

**Figure 4:**
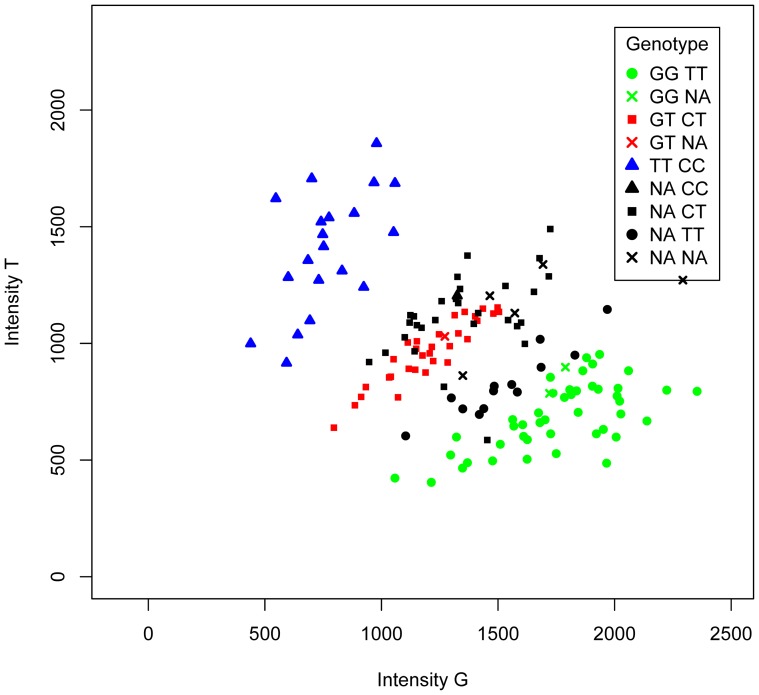
Intensity plot of a G/T polymorphism for 146 individuals. Colours indicate the genotype of the G/T polymorphism to be imputed, symbols indicate the genotype of the G/T and a covariate C/T polymorphism.

Table 2 shows the different estimates of the inbreeding coefficient, together with their confidence intervals and *p*-values for a HWP test. Missing data statistics are also shown. Statistic 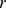 is the relative increase in the variance of the inbreeding coefficient due to missings. The lowest values of 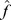 are obtained for models using SNPs as covariates. Statistic 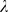 is termed the fraction of missing information about the inbreeding coefficient. These standard missing-data statistics quantify to what extent the standard errors of the inbreeding coefficient are affected by missing data. The fraction of missing information quantifies how much of the sampling variance of the inbreeding coefficient can be ascribed to missing data. The first “model” in Table 2 consisted of just discarding missings and gave a significant chi-square statistic in a test for HWP (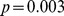). Model 2 used imputation by taking a random sample of the observed data, and corresponds to assuming MCAR. This yields, as expected, an inbreeding coefficient that is close to the one with missings discarded, but has the advantage of providing an estimate of the fraction of missing information, showing that 22% of the sampling variance of 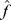 is due to missing data. For models with covariate SNPs only 3 through 4% of the sampling variance of 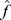 is attributable to missing data, and this is five times less than a model using intensities only. This suggests covariate SNPs should be included in the imputation model, as they mitigate the effect of missing data on the estimation of 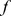. Table 2 shows that imputation of the missings with the aid of the intensities (model 3) renders the deviation from HWP insignificant (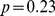). When a correlated flanking marker, a C/T polymorphism (model 5) is included in the model, the inbreeding coefficient drops down to 0.060, and becomes even less significant (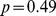). Figure 4 shows the same intensity plot of the G/T polymorphism as represented in Figure 1, but now symbols indicate the genotype of the correlated covariate. The plot shows that genetic covariates can be helpful in classifying the missing values. Most missings on top of the heterozygote cloud are apparently heterozygotes with respect to the response, based on their correlated covariate value of CT, which tends to correspond to GT heterozygotes in the observed data. Most missings on top of the GG cloud are seen to be GG genotypes, based on their correlated covariate value of TT, which tends to correspond to GG in the observed data. This inference is possible thanks to the correlation between response and covariate SNPs (linkage disequilibrium). Additional covariates, whether intensities or SNPs may be helpful to classify the remaining “double missings” (cases with missings for the GT and the TC polymorphism) or to improve the classification of the “single missings” of the GT polymorphism. The distribution of the genotypes for this correlated marker differed for missings and non-missings of the SNP to be imputed (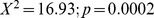) indicating the MCAR assumption does not hold w.r.t. this correlated SNP. The inclusion of 9 additional correlated SNPs (models 6 and 7 in Table 2 does not substantially alter the conclusion, and provided approximately the same estimate and confidence intervals for 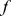. All computations in Table 2 were repeated using Fisher's 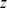-transformation for the inbreeding coefficient. Results with Fisher's transformation were almost identical to those given in Table 2. All models based on allele intensities and correlated markers in Table 2 show lower estimates of the inbreeding coefficient, and clearly indicate that there is no evidence for rejecting HWP for this SNP. The results of multiple imputation shown in Figure 3 reveal that the multinomial logit models basically impute heterozygotes for the missing values. We assessed the convergence of the MICE algorithm by making plots of the inbreeding coefficient against the iteration number (see Figure S1). These plots showed good mixing and no trends, suggesting that the algorithm had converged.

### Multiple imputation for a set of SNPs

**Figure 5:**
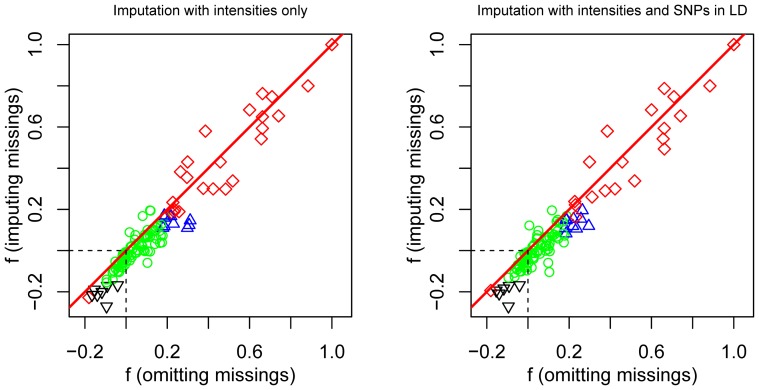
Estimation of inbreeding coefficients by multiple imputation and by omitting missings. Left panel: using allele intensities only. Right panel: using allele intensities and covariate SNPs in LD (complete and incomplete) with 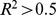. Symbols indicate the result of two significance tests: a test for HWP discarding missings and a test for HWP with imputation of missings. Circles: SNPs with both tests non-significant; Diamonds: SNPs with both tests significant; Upward triangles: SNPs with a significant chi-square test when missings are omitted, but an insignificant test when missings are imputed. Downward triangles: SNPs with a non-significant chi-square test when missings are omitted, but a significant test when missings are imputed.

The procedure outlined above was repeated for the set of 140 SNPs with 10–50% missing values where we imputed SNPs with missings in a one-by-one manner, using five models with different predictors. The first model (A) used only allele intensities for imputation. The second model (B) used allele intensities and completely observed SNPs that were in linkage disequilibrium (LD) with the SNP with missings. The third model (C) used completely observed SNPs in LD only. The fourth model (D) used intensities and SNPs (like model B), but allowed the explanatory SNPs to have missing observations as well. The fifth model (E) uses only SNPs, which can be complete or incomplete. This last model is probably the most useful in practice, since intensities are not always available, and correlated flanking SNPs typically have missings as well. Covariate SNPs were included in the model as a predictor when their 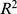 statistic for LD with the response SNP was above 0.5. This criterion implied that there were on average 1 or 2 covariate SNPs in the models B and C, and more in models D and E. If no SNPs satisfied the 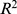 criterion in models B and D, then imputation was carried out with allele intensities only. Figure 5 shows the relationship between the inbreeding coefficients obtained by discarding missings and by imputing missings for two of the five models, models A and D with the largest and smallest median fractions of missing information.

Each SNP was tested twice for HWP (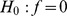 against 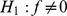). The first test discarded missings and the second test used multiple imputation of missings. The vast majority of the SNPs has a positive inbreeding coefficient (lack of heterozygotes) that drops when missings are imputed, as most SNPs in Figure 5 fall below the 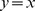 line. This means that missings are relatively more often imputed as heterozygotes. Most SNPs are jointly non-significant in both tests. Two sets of boundary SNPs were found. One set with a positive inbreeding coefficient (upward triangles in Figure 5 that appears significant in a chi-square test with omission of missings, but non-significant after imputation of the missings, and a second set with the reverse condition (downward triangles, significant deviation from HWP under imputation, non-significant deviation under discarding of missings). Table 3 summarizes test results and fractions of missing information for the five models considered. From a qualitative point of view multiple imputation changed the inference about HWP considerably: for 10 to 17% (depending on the model) the test result was reversed with respect to a test that discarded missings. The models that impute with only SNPs as covariates (C,E) showed less evidence for deviation from HWP. The overall percentage of significant SNPs as judged by a chi-square test without imputation was 27%.

**Table 3:** Number of imputed SNPs, number and percentage of significant SNPs with missings imputed, mean, median and maximum of the fraction of missing information (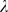) for multinomial logit models with five different sets of predictors.

Model	# SNPs	# sign.	% sign.	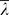	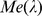	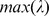	% reversal
A. Intensities	140	36	25.7	0.166	0.111	0.770	11.4
B. Intensities and complete SNPs	140	34	24.3	0.132	0.058	0.553	12.9
C. Complete SNPs	69	11	15.9	0.071	0.044	0.436	10.1
D. Intensities and SNPs	126	30	23.8	0.122	0.040	0.582	12.7
E. SNPs	78	11	14.1	0.079	0.043	0.436	16.7
IMPUTE2	140	24	17.1	-	-	-	17.1

The last column (% reversal) indicates the percentage of SNPs whose test results changed status (from significant to non-significant or the reverse) in comparison with a test omitting missings.

### Comparison with imputation using a reference panel

**Figure 6:**
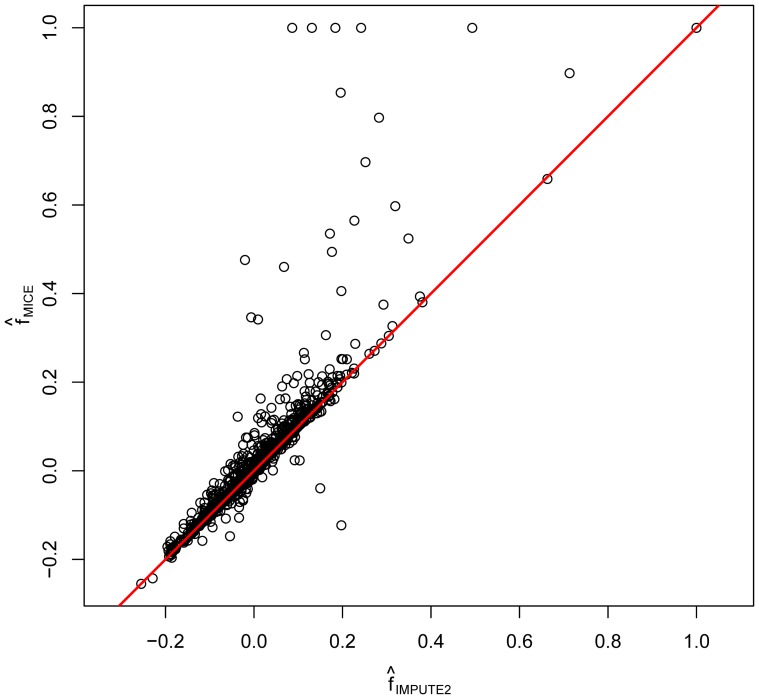
Scatter plot of inbreeding coefficients for 1070 non-monomorphic SNPs with missings obtained by multiple imputation (MICE) and single imputation (IMPUTE2).

When GWAS or fine-mapping genotype data is available, missing genotype information is often imputed using an external reference panel, and this exploits known LD structure. The programs IMPUTE [24] and MaCH [25] are, among others, based on this principle. We used IMPUTE2 as a single-imputation method, after prephasing the data with the program SHAPEIT [26]. Inbreeding coefficients were calculated after the genotype data had been completed this way. A plot of the inbreeding coefficients obtained by multiple imputation with MICE against the inbreeding coefficients obtained after imputation by IMPUTE2 is shown for the set of 1070 non-monomorphic SNPs with missings in Figure 6. The multinomial logit model in MICE used allele intensities and 4 flanking SNPs as covariates. Both estimates correlate well (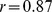). Note that for some outlying markers multiple imputation with MICE yielded an estimate of 1, whereas the corresponding IMPUTE2 estimates were much lower. For a few markers MICE gave considerably lower inbreeding coefficients (See the discussion for these issues.)

We have carried out a simulation study in order to further compare single imputation by IMPUTE2 and multiple imputation by MICE. For this purpose we selected the 504 SNPs of the database that had no missing genotypes and that were not monomorphic. From this complete database we deleted genotypes according to two different missing data mechanisms, MCAR and MNAR. We then imputed missings using multiple imputation by chained equations with MICE and using a multinomial logit model that used the two allele intensities and 4 flanking SNPs as covariates. Missings were also imputed using IMPUTE2. We computed the root mean squared error in the inbreeding coefficient 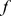 for both the multiple and single imputation method, as well as for the computation of 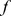 with missings discarded. Genotypes were deleted by randomly selecting markers, and selectively deleting genotypes according to a given vector of probabilities shown in Table 4. When the three probabilities are equal for the three genotypes, the missing data mechanism is MCAR, if not, it is MNAR. Table 4 shows that for IMPUTE2 the RMSE is always zero. IMPUTE2 apparently infers the missing genotype data without error from the reference panel, and thus the estimated inbreeding coefficient after imputation by IMPUTE2 is the same as the inbreeding coefficient for the complete data. For multiple imputation with MICE, the RMSE is generally small, but increases if there is severe disequilibrium (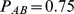). If the data is MNAR, then discarding the missings gives the worst estimates of 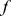.

**Table 4:** Simulation results.

			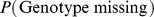			RMSE		
Regime	% missing	% SNPs	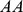	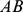	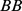	DISCARDING	MICE	IMPUTE2
MCAR	6	25	0.25	0.25	0.25	0.01	0.03	0.00
	12	50	0.25	0.25	0.25	0.01	0.03	0.00
	19	75	0.25	0.25	0.25	0.01	0.04	0.00
MNAR	3	25	0.05	0.25	0.05	0.08	0.03	0.00
	4	25	0.05	0.50	0.05	0.21	0.07	0.00
	6	25	0.05	0.75	0.05	0.42	0.17	0.00
	5	50	0.05	0.25	0.05	0.08	0.03	0.00
	9	50	0.05	0.50	0.05	0.21	0.08	0.00
	13	50	0.05	0.75	0.05	0.43	0.17	0.00
	8	75	0.05	0.25	0.05	0.08	0.04	0.00
	14	75	0.05	0.50	0.05	0.21	0.10	0.00
	19	75	0.05	0.75	0.05	0.43	0.23	0.00

Overall percentage of missing data, percentage of SNPs with missings, probabilities of missingness for the three genotypes and the root mean squared error (RSME) for the inbreeding coefficient (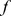) when missings are discarded, imputed by MICE or imputed by IMPUTE2, under MCAR and MNAR.

## Discussion

Testing genetic markers for HWP is a standard aspect of the statistical analysis of polymorphisms involved in genetic association studies. Missing values are typically ignored in tests for HWP, and this can lead to biased inference about HWP as shown by the example in the Results section. For the data studied in this paper, extracted from a real genotyping study similar to most GWAS performed to date, missing genotypes can definitely not be considered missing completely at random. Imputation of missing genotype information can then improve the inference for HWP. We propose to use a general multiple imputation procedure based on a multinomial logit model that can incorporate information from allele intensities and neighbouring SNPs, if available. This approach does not require dense SNP genotyping typical of a GWAS study. For the latter, imputation based on reference panels can be even more efficient to recover missing genotypes and avoid biased estimates of HWP.

The allele intensities and correlated flanking markers are strong predictors for imputing a polymorphism with missing values. The proposed multinomial logit model used often showed perfect separation (in that case the genotypes of a marker can be predicted without error from intensities or correlated markers). This phenomenon is described in the context of logistic regression by Agresti [16]. Estimated standard errors of the predictors tend to be very large in models with perfect separation, leading them to be “insignificant”. This is a numerical problem, and by no means implies the predictors are useless for imputations.

This paper shows how to perform inference for HWP in the presence of missing data by multiple imputation, using the inbreeding coefficient. This approach is closely related to the use of the classical chi-square test as a tool for testing for HWP. Over the last decade, the exact test for HWP has become more popular. In ongoing research we evaluate inference for HWP in the presence of missings by combining exact test results of imputed data sets. The EM algorithm could be used as an alternative way for estimation of the inbreeding coefficient in the presence of missings. This does however, not readily provide standard errors for the estimates.

The two imputation methods used in this paper both have their pros and cons, which we briefly discuss. The MICE algorithm is versatile tool allowing us to test for HWE in the presence of missing data. The algorithm is not limited to genetic marker information but can use all kind of covariates that may be available for imputation (allele intensities, metabolites, physiological variables, etc.). Only a few informative covariates are needed in order to improve inference for HWE and correct the bias that would be caused by discarding the missings. The method implemented in IMPUTE2 relies on reference panels of extensive genetic information, and so requires and uses much more information than MICE. In this respect it is no surprise that IMPUTE2 outperforms MICE in the simulations. Most likely, the RMSE for the MICE estimates could be decreased by including more genetic covariates, though this would slow down the computations. On the other hand, a limitation of the MICE program is that it cannot impute categories that are not present in the sample data. This means that markers with a low MAF for which no heterozygotes are observed in the data, missings will never be imputed as heterozygotes. In these circumstances the program will basically impute the most common homozygote, leading to an estimated inbreeding coefficient of 1. This is the explanation for the appearance of some MICE estimates that have 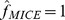 whereas the corresponding estimates obtained by IMPUTE2 are much lower. Likewise, the markers for which MICE gave considerably lower inbreeding coefficients in comparison with IMPUTE2 correspond to SNPs for which one of the homozygote counts is zero. This gives a negative inbreeding coefficient.

Table 3 shows that models C, D and E had a lower number of imputable SNPs. This was due to the fact that the MICE algorithm was not always able to create imputed data sets. This occurred when the predictor was perfectly related to the response (a diagonal contingency table) or when there was strong collinearity between predictor SNPs. Imputation for models C and E was neither possible when there were no predictor SNPs in LD with 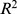 below 0.5. These problems typically occurred with SNPs with a low minor allele frequency that lead to sparse contingency tables.

In this paper we have made no distinction between cases and controls. In principle one would expect more disequilibrium for cases, due to possibly different survival rates of affected genotypes. However, the sample sizes needed to detect disease association effects are very large [11]. The data set in this study is probably too small to detect deviations from HWP due to disease association. The excess of significant SNPs found in the Results section is most likely explained by some genotyping error. We note in this respect that the 140 SNPs studied in the Results section had very similar rates of significant SNPs for cases and controls (21% versus 17% respectively, with 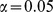), even though the sample size of the cases doubled that of the controls.

Population genetic textbooks [27], [28] typically point out that Hardy-Weinberg equilibrium will be observed if a long list of assumptions is met (random mating, no selection, no mutation, etc.). The interpretation of HWP test results often varies depending on the context of the study. Rejection of HWP is often explained as follows: 1) a chance effect (especially if many markers are tested), 2) evidence for the existence of genotyping error, 3) evidence for the existence of marker-disease association, 4) evidence for selection, 5) existence of population substructure (the sample is a non-homogeneous population [29]) or 6) violation of one (or more) other assumptions underlying HWP. The results in this paper show that the latter list should be extended with an additional consideration: rejection occurred because a considerable part of the observations were missing (possibly related to genotyping error or wrong genotype calling), and these observations were discarded prior to testing for HWP. This phenomenon may have been relevant since the earliest tests for HWP with genetic markers up till the massive use of these tests in genome-wide association studies today.

## Availability of Software and Data

A function for performing tests for HWP that takes missing data into account by multiple imputation is available in the R-package HardyWeinberg [30]. The function takes a SNP with missing values as its main argument, and covariates that can be used for imputation (intensities, flanking or correlated markers) can be supplied. The multiple imputation part is done by the R-package MICE [18].

The genetic data used in this study can be made available upon request, but will be subject to a written guarantee of confidentiality.

## Supporting Information

Figure S1 **Convergence plots of the inbreeding coefficients for five models using MICE from** **Table 2** **.**
(TIF)
